# Association of SNPs in *EGR3* and *ARC* with Schizophrenia Supports a Biological Pathway for Schizophrenia Risk

**DOI:** 10.1371/journal.pone.0135076

**Published:** 2015-10-16

**Authors:** Matthew J. Huentelman, Leela Muppana, Jason J. Corneveaux, Valentin Dinu, Jeremy J. Pruzin, Rebecca Reiman, Cassie N. Borish, Matt De Both, Amber Ahmed, Alexandre Todorov, C. Robert Cloninger, Rui Zhang, Jie Ma, Amelia L. Gallitano

**Affiliations:** 1 Neurogenomics Division, Translational Genomics Research Institute, Phoenix, Arizona, United States of America; 2 Department of Basic Medical Sciences, University of Arizona College of Medicine—Phoenix, Phoenix, Arizona, United States of America; 3 Department of Biomedical Informatics, Arizona State University, Scottsdale, Arizona, United States of America; 4 Department of Psychiatry, Washington University School of Medicine, St. Louis, Missouri, United States of America; 5 Xi’an Hong Hui Hospital, the Affiliated Hospital of Xi’an Jiaotong University School of Medicine, Xi’an, Shaanxi, China; 6 Department of Biochemistry and Molecular Biology, Xi’an Jiaotong University School of Medicine, Xi’an, Shaanxi, China; Kunming Institute of Zoology, Chinese Academy of Sciences, CHINA

## Abstract

We have previously hypothesized a biological pathway of activity-dependent synaptic plasticity proteins that addresses the dual genetic and environmental contributions to schizophrenia. Accordingly, variations in the immediate early gene *EGR3*, and its target *ARC*, should influence schizophrenia susceptibility. We used a pooled Next-Generation Sequencing approach to identify variants across these genes in U.S. populations of European (EU) and African (AA) descent. Three *EGR3* and one *ARC* SNP were selected and genotyped for validation, and three SNPs were tested for association in a replication cohort. In the EU group of 386 schizophrenia cases and 150 controls *EGR3* SNP rs1877670 and *ARC* SNP rs35900184 showed significant associations (p = 0.0078 and p = 0.0275, respectively). In the AA group of 185 cases and 50 controls, only the *ARC* SNP revealed significant association (p = 0.0448). The *ARC* SNP did not show association in the Han Chinese (CH) population. However, combining the EU, AA, and CH groups revealed a highly significant association of *ARC* SNP rs35900184 (p = 2.353 x 10^−7^; OR [95% CI] = 1.54 [1.310–1.820]). These findings support previously reported associations between *EGR3* and schizophrenia. Moreover, this is the first report associating an *ARC* SNP with schizophrenia and supports recent large-scale GWAS findings implicating the ARC complex in schizophrenia risk. These results support the need for further investigation of the proposed pathway of environmentally responsive, synaptic plasticity-related, schizophrenia genes.

## Introduction

Schizophrenia is a severe mental illness that affects approximately 1% of the world’s population and is determined by both genetic and environmental factors [1, 2]. The genetic contribution to schizophrenia risk ranges from 50–80% [1, 3]. The remaining influence may be attributed to environmental factors [2]. However, *how* environmental exposures interact with genetic variations to influence schizophrenia susceptibility remains unclear.

Immediate early gene (IEG) transcription factors are rapidly activated in the brain in response to environmental stimuli and regulate downstream neuronal gene expression. The early growth response gene (*EGR*) family of IEG transcription factors is involved in numerous neural processes, including regulation of synaptic proteins and synaptic plasticity, dysfunction of which have been hypothesized to play a role in schizophrenia pathogenesis [4–10].

Numerous findings suggest a role for the IEG *EGR3* in schizophrenia susceptibility. Single nucleotide polymorphisms (SNPs) in *EGR3* are associated with schizophrenia in Japanese, Korean, and Han Chinese (CH) populations [11–13], and levels of *EGR3* mRNA are reduced in post-mortem brain tissue from schizophrenia patients [11, 14]. We have previously reported that *Egr3*-deficient (*Egr3*KO) mice display schizophrenia-like behavioral abnormalities that are reversible with antipsychotic treatment [4, 15]. *EGR3* is regulated downstream of several key schizophrenia candidate proteins, including Neuregulin 1, N-methyl D-Aspartate Receptors (NMDARs) and calcineurin (CN) [11, 16–21], and *Egr3*KO mice share key phenotypes with NMDARKO and CNKO mice, including memory dysfunction and deficits in the form of hippocampal synaptic plasticity long-term depression (LTD) [4, 22, 23]. These shared characteristics suggested to us that these proteins may form a biological pathway of genes which, when disrupted may increase risk for schizophrenia. If so, then dysfunction of the gene activity-regulated cytoskeleton-associated protein (*ARC*) [24], which is a downstream target of *EGR3* and is similarly required for memory formation and hippocampal LTD in mice [25–27], may also confer risk for schizophrenia.

In support of this hypothesis, several large-scale studies have identified associations between schizophrenia and genes whose proteins complex with ARC in the post-synaptic density of neurons [28–30]. However, to date there have been no reports of genetic association between SNPs in the *ARC* gene itself and schizophrenia. In addition, significant associations between SNPs in *EGR3* and schizophrenia have not been reported in populations of European or African descent. We therefore carried out the current studies to test the hypothesis that *EGR3* and *ARC* are schizophrenia risk associated genes.

## Materials and Methods

### DNA Samples

DNAs from EU and AA patients diagnosed with schizophrenia based on DSM-IV classification were obtained from the National Institute of Mental Health Schizophrenia Genetics Initiative (NIMH-SGI) Release 1.0 and Release 3.0. Control samples were obtained from the NIMH-GI Control Database Release 3.0 and the Environmental Catchment Area ADHD Study. Additional control genotypes were obtained from the 1,000 genomes published findings [31]. Written informed consent was obtained from subjects in all studies. These samples were obtained in a de-identified manner and were thus exempt from approval by the University of Arizona Institutional Review Board (IRB).

The study involving CH samples was approved by the genetic research ethics committee (equivalent to the IRB) of Xi’an Jiaotong University School of Medicine and written informed consent was obtained from all subjects. A total of 982 subjects included 491 schizophrenics (253 males, mean age = 34.49±11.85, age of onset = 24.30±7.22; 238 females, mean age = 31.77±13.50, age of onset = 24.42±8.64) and 491 normal controls (272 males, mean age = 28.92±13.78; 219 females, mean age = 28.88±13.83). All patients were diagnosed by the psychiatrists of the First Affiliated Hospital of Xi’an Jiaotong University School of Medicine according to *Diagnostic and Statistical Manual of Mental Disorders* (DSM-IV) criteria for schizophrenia using a combination of examination of psychiatric case records and clinical interview. The diagnosis was checked and verified by two independent senior psychiatrists who reviewed the psychiatric case records.

CH controls were drawn from a combination of local volunteers or blood transfusion donors. Subjects with current or past evidence of mental illness, or with a first-degree relative with mental illness, were not included in the cohort. In addition, in China only healthy people not taking medication can donate blood, hence the evaluated controls were medication-free. Overall, these controls are unlikely to include a significant number of psychotic individuals, if indeed any. All subjects were *Han* Chinese in origin and from Northwest China.

### Next-Generation Sequencing (NGS)

#### Amplification and sample preparation

Pooled DNA samples were PCR amplified using RainStorm (RainDance Technologies, Inc. Billerica, MA) essentially as described in [32]. Briefly, individual DNA samples were quantitated in triplicate with PicoGreen reagent (Life Technologies, Grand Island, NY) and equimolar pools were created based on sex, race, and diagnosis. Individual DNAs were fragmented to 2–4 kb lengths and merged with a panel of primers designed to create overlapping amplicons spanning the entire coding and noncoding sequence of *EGR3* and *ARC*, from 7 Kb upstream of the 5’UTR to 3 Kb downstream of the 3’UTR. See S1 Table for primer sequences. RainStorm PCR was performed and amplification products were concatenated using the New England Biolabs (NEB) Quick blunting and ligation kit (Ipswich, MA).

Amplicons were sonicated for 2.5 minutes to produce 100–400 bp DNA fragments, treated with Klenow enzyme Fragment for 30 minutes, blunt-ended with T4 DNA polymerase and T4 polynucleotide kinase, and purified using the Ampure SPRI bead purification system (Beckman Coulter, CA). Fragments were 3’ end-labeled with an untemplated A base (Klenow 3’-5’ exo- and dATP for 30’) followed by Ampure purification and PicoGreen quantification (Invitrogen, Inc., NY). NGS universal adapters were modified with unique DNA bar-codes as previously described [33]. Ligation was performed using 10:1 molar ratio of bar-coded adapters:DNA fragments using high concentration T4 DNA ligase for 15’. The ligation product underwent gel purification (run on a 3% agarose gel at 100 v for 2 h, the 300 bp band was excised and purified using Freeze’ N Squeeze (Bio-Rad Laboratories, Inc., CA) columns) and PCR amplification. A second round of gel purification isolated the 200bp-350bp fragment to form a DNA library bar-coded for each individual DNA sample.

#### NGS of pooled bar-coded DNAs

Individual bar-coded DNA libraries were combined into pools and added to a sequencing flowcell to generate clusters that were subjected to NGS. The molecular bar-coding approach facilitated sequencing of DNA samples from multiple pools on each lane of the flowcell.

#### NGS data analysis and variant detection

Data from the Illumina Genome Analyzer was converted to text-based sequence with the Illumina Pipeline 1.4 and CASAVA 1.0 software using standard Illumina protocols. Image cluster identification, sharpening, background subtraction, and intensity extraction were performed with the Firecrest module. Base calling and phasing/prephasing effects were corrected for using the Bustard module. Using the pass/fail filter generated by Bustard, Sanger Phred-33 FASTQ files were created with a custom perl script in preparation for alignment. FASTQ format was chosen because each sequence read is described with the sequence and individual base quality scores, metadata from the instrument, cluster position and paired-read info, and many of the commonly used aligners use the FASTQ format.

NGS data were aligned with the reference genome using the BWA aligner [34], the tool used by the 1000 Genomes project, which uses SAM (Sequence Alignment/Map) format [35]. The SAM format contains information on mapping quality, mapping coordinates, mismatches, and fields for user specified metadata. BWA alignment was carried out using default modes for 36 bp single and paired read (72 bp total) runs. Reads were mapped to the reference human genome used by the 1000 Genomes project [31]. After alignment, variants were called with `VarScan.v2.2.jar pileup2snp`with the options `—min-coverage 250—min-reads2 10—min-avg-qual 20—min-var-freq 0.02—p-value 0.01`.

### Genotyping of selected SNPs

DNA samples from the NGS discovery cohort were genotyped for three *EGR3* SNPs and one *ARC* SNP for the validation phase association analyses. Additional schizophrenia case DNAs for the replication association study were obtained from NIMH-SGI release 3.0. DNAs, were quantitated by nanodrop spectrophotometry, diluted in sterile Tris buffer, 10mM, pH 7.4, to a concentration of 4 ng/ul, and confirmed using PicoGreen (Invitrogen, Inc., NY) quantification, according to the manufacturer’s protocol. Samples were plated on to 96 well plates at 20 ng/ well, and dried down overnight at room temperature in the dark. DNAs were genotyped using individual SNP assays from Applied Biosystems Inc., (ABI, Foster City, CA) on an ABI 7500 Fast QRT-PCR instrument following the manufacturer’s protocol.

Genotyping of CH samples was performed using direct DNA sequence analysis. The PCR primers for amplification of the rs35900184 were as follows: forward primer 5’- CGCCTGGAGAAGAATCAGAG-3’ and reverse primer 5’- AAAGACTTTGTGGGAACCTTGA-3’. The PCR reaction was performed in 25 μl of standard PCR buffer containing 1.5mM MgCl_2_, 0.2mM of each dNTP, 0.5μl of each primer, 1 unit of Taq DNA polymerase, and 25 ng of human genomic DNA. The program was one cycle of 2min for denaturation at 95°C, 35 cycles of 30s at °C, 35s at 58°C, 45s at 72°C, and one 7min extension step at 72°C. Purified PCR products were sequenced bi-directionally using PCR primers as sequencing primers and the Applied Biosystems Prism BigDye terminator cycle sequencing reaction kit. The products were evaluated on an ABI 3730 Genetic Analyzer (Applied Biosystems).

### Individual Genotype Analysis

Genotype data were analyzed using PLINK v1.07 [36], and the Fisher test with 95% confidence intervals.

### Linkage Disequilibrium (LD) Analyses

Haploview [37] was used to estimate LD between SNPs across the *ARC* region in the CEPH population (Utah residents with ancestry from northern and western Europe, abbreviated CEU), Hapmap [38] data release 28. The *ARC* region (chromosome 8, from 143,689,412 to 143,692,835) included 18 SNPs in the CEU Hapmap individuals. Hapmap Genome Browser release 28 (http://hapmap.ncbi.nlm.nih.gov/) with Copy Number Variation Region Track enabled, was used to identify known CNVs in the *ARC* region on chromosome 8.

### Power Analyses

Calculating power for genetic studies requires knowledge of values such as the prevalence of the disease, the relative risk of the disease risk allele (the ratio of the probability of disease occurrence in the risk-allele carriers to the probability of disease occurrence in individuals that are not carriers of the risk-allele) and the frequency of the disease allele. Since these parameters are unknown for the current study, estimates were used, and the disease risk allele was assumed to be included in our sample. Assuming values of 0.01 for disease prevalence, relative-risk of 1.5 (a relatively optimistic value for a complex disease such as schizophrenia), alpha-level of 0.05 and a standard 1df allelic test, then the estimated power to detect a significant association ranges from 0.05 to 0.47 (for disease allele frequencies ranging from 0.1 to 0.9) for a sample size of 386 cases and 150 controls, as was our EU cohort. For our AA cohort (sample size of 185 and 50 controls) the power range is lower, from 0.05 to 0.21 only. These parameter ranges indicate that our sample size of fewer than 200 cases and 50 controls is underpowered to detect an association in the SNPs in the AA cohort, should one truly exist. Increasing the number of cases and controls to N = 1,200, and 300, respectively, would increase the power to detect a positive association to 0.80, for disease allele frequencies of 0.2 (which approximates the MAF for rs1877670 in AA). The Genetic Power Calculator was used to generate these power estimates [39].

## Results

Since the frequencies of specific SNPs, as well as their potential association with an illness, vary among ethic and racial groups, it can be difficult to select the SNPs that have a high likelihood of showing association within the population of study. In studies with limited sample sizes limiting the number of SNPs queried helps to maintain statistical power. To address these issues we employed a method using NGS to identify all variations across the *EGR3* and *ARC* loci from a discovery cohort of pooled DNA samples. SNPs in which one allele demonstrates a high degree of difference in frequency between cases and controls, particularly in two separate populations, may be more likely to show association with illness.

### NGS of the *EGR3* and *ARC* loci

To identify variations within the *EGR3* and *ARC* loci that have a high likelihood for association with schizophrenia risk in two different racial groups, we used a two-step approach. For the initial step we used a “Discovery” cohort of DNA samples from schizophrenia patients (cases) and non-psychiatrically ill individuals (controls) that were matched for sex and race (EU and AA). Table 1 shows how these samples were pooled into 16 groups of up to 25 samples per group. NGS across the *EGR3* and *ARC* loci was performed on each pool and results were compared among pools for quality control.

**Table 1:** Characteristics of DNA Pools Used for Next Generation Sequencing.

Race	Sex	Affected Status	No. of Subjects/Pool	No. of Pools	Total No. of Subjects
**EU**	F	Cases	25	2	50
	F	Controls	25	2	50
	M	Cases	23–25	4	95
	M	Controls	25	4	100
**AA**	F	Cases	18	1	18
	F	Controls	25	1	25
	M	Cases	24	1	24
	M	Controls	25	1	25

Allele frequencies were estimated for each variant along the entire sequenced region in each pooled group using CRISP [40]. The results were averaged among pools according to case/control status and race (sex was not maintained as a variable). For each variant the difference in the average frequency of the minor allele between cases and controls, termed the “delta Minor Allele Frequency” (Δ MAF), was calculated by subtracting the average MAF for schizophrenia patients from that for controls of the same race. S2 and S3 Tables show the allele frequency results for all variations identified in the *EGR3* region, ranked from highest to lowest Δ MAF, in the EU and AA groups. S4 and S5 Tables show the same analysis for the sequenced *ARC* region in the respective EU and AA groups.

**Fig 1:**
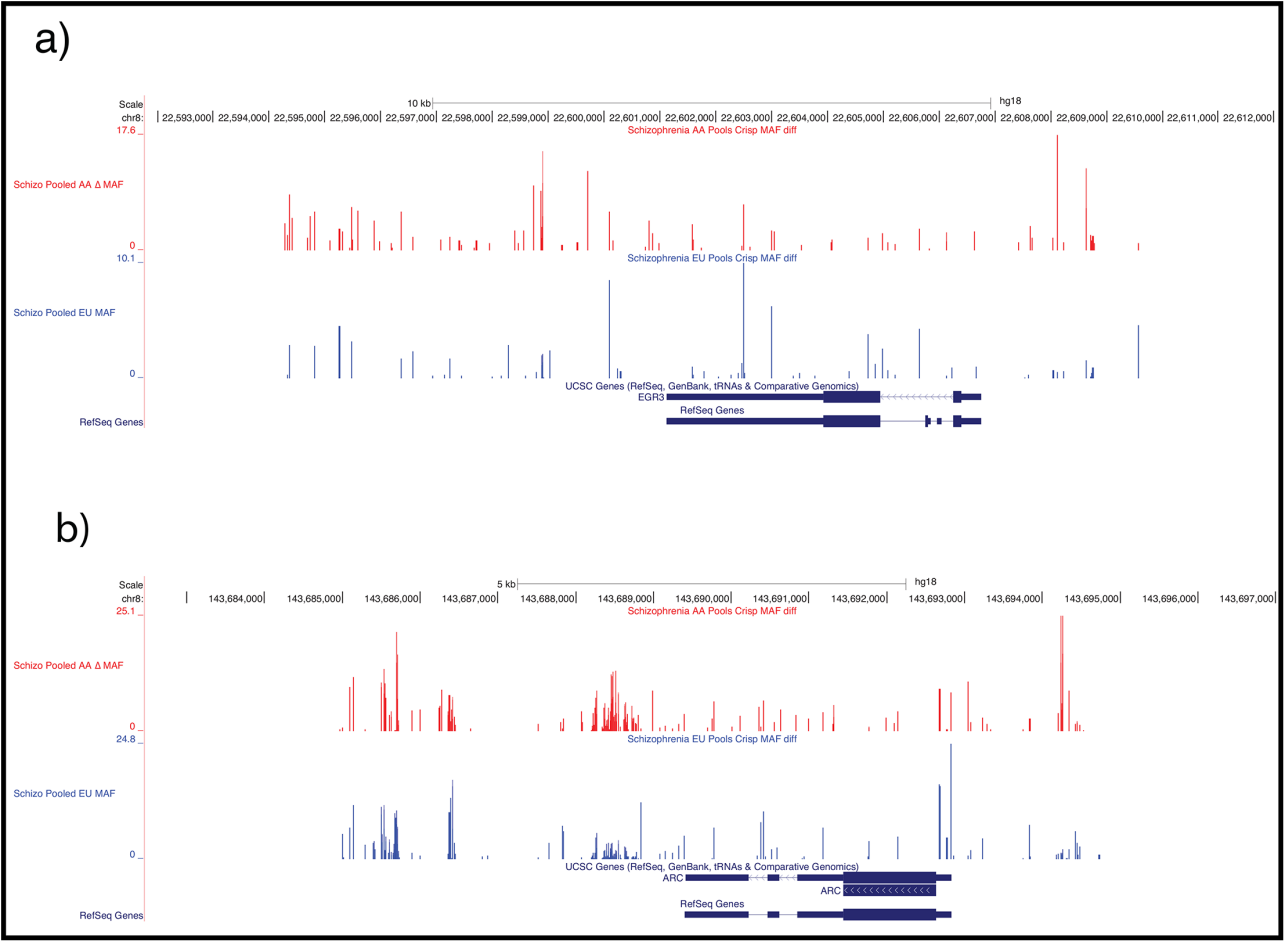
Minor Allele Frequency Differences in EU and AA. Results of polymorphisms identified by NGS are mapped to the reference human genome used by the 1000 Genomes project (hg18). Vertical lines indicate the Δ MAF (difference between the minor allele frequency in cases versus controls) for each base pair of the genome region investigated. Higher peaks indicate a greater difference in prevalence of that variation between cases and controls, and thus a higher potential for association with schizophrenia risk. The red graph indicates data for Whites and the Blue graph indicates data for African Americans. (A) Δ MAF map for the *EGR3* locus, hg18 coordinates chr8:22,591,791–22,612,066. (B) Δ MAF map for the *ARC* locus, hg18 coordinates chr8:143,682,474–143,697,026

The Δ MAF results for the sequenced regions of the EU (in blue) and AA (in red) populations were plotted onto the genome map in the regions of *EGR3* (Fig 1A) and *ARC* (Fig 1B) using the UCSC genome browser. Individual variations that produced high peaks (i.e. greater Δ MAF) in both racial groups were of greatest interest.

### Case-Control Genetic Association Analyses

Several SNPs were selected for validation of the NGS results by genotyping of the individual DNA samples from the discovery cohort. Variations for genotyping validation were selected based on a combination of criteria, including high Δ MAF values in both race cohorts and/or having been reported as associated with schizophrenia in other populations. Three SNPs from the *EGR3* region were selected. The SNP **rs1877670** displayed the highest Δ MAFs in our EU cohort, and ranked eighth in the AA cohort, with a Δ MAF of 7.0%. This SNP was also reported in a haplotype associated with schizophrenia in a CH population [41]. **rs1996147** demonstrated Δ MAFs of 8.6% in EUs and 5.9% in AAs in our NGS discovery analysis, and showed nominally significant association with bipolar disorder in a study of circadian rhythm genes [42]. The SNP **rs10095121** showed a Δ MAFs of 2.9% in EUs and 8.4% in AAs in our NGS results, and revealed nominally significant association with the diagnosis of child bipolar I disorder in our prior family based association study [43].

Since no studies had been published investigating *ARC* SNPs for association with schizophrenia, selection of a single *ARC* SNP was made based only on high Δ MAF values in both race cohorts. The SNP rs28420666 was initially selected for genotyping as it demonstrated Δ MAF of 24.8% in the EU cohort 8.3% in the AA cohort. However our attempts to create a genotyping assay for this SNP were unsuccessful. Therefore SNP **rs35900184,** which showed Δ MAFs of 10.2% in EUs and 6.6% in AAs, was selected.

Tables 2 and 3 show the results of the validation phase. Of note, the pooled sequencing approach yielded MAFs that were similar to the confirmed individual-based results (e.g. comparing MAF Controls versus MAF Pooled Controls columns in Tables 2 and 3). In the EU group *EGR3* SNP rs1877670 showed a trend toward association (p = 0.07), and the *ARC* SNP rs35900184 revealed a significant association with the schizophrenia diagnosis (p = 0.02). In the AA cohort, there were no significant associations between the three *EGR3* or the single *ARC* SNP tested, though the *ARC* SNP rs1996147 showed a trend toward significance (p = 0.07). We selected the three SNPs that showed significance or trended toward significance for further evaluation in a replication group.

**Table 2:** Validation Phase: Comparison of Pooled Sample NGS with Individual Genotyping Results and Genetic Association Analyses in EU cohort. Pooled EU.

SNP	Chr	Hg18 Position	A1	A2	MAF Controls	MAF Cases	MAF Pooled Controls	MAF Pooled Cases	GMAF	P-value	OR [95% CI]
rs10095121	8	22,538,426	C	T	33.6%	32.8%	32.6%	29.8%	28.4%	0.8566	0.970 [0.680–1.380]
rs1877670	8	22,546,561	C	T	45.8%	54.1%	42.3%	47.6%	44.3%	0.0685	1.390 [0.990–1.960]
rs1996147	8	22,544,158	G	A	35.5%	32.7%	33.5%	25.0%	41.2%	0.5307	0.890 [0.620–1.260]
rs35900184	8	143,693,411	T	C	22.9%	32.3%	18.0%	28.1%	26.5%	0.0215	1.610 [1.090–2.390]

*145 cases*, *150 controls; 195 males*, *100 females*.

**Table 3:** Validation Phase: Comparison of Pooled Sample NGS with Individual Genotyping Results and Genetic Association Analyses in AA cohort. Pooled AA.

SNP	Chr	Hg18 Position	A1	A2	MAF Controls	MAF Cases	MAF Pooled Controls	MAF Pooled Cases	GMAF	P-value	OR [95% CI]
rs10095121	8	22,538,426	C	T	26.1%	24.4%	31.8%	20.0%	28.4%	0.8621	0.910 [0.460–1.820]
rs1877670	8	22,546,561	C	T	22.3%	14.6%	29.0%	19.0%	44.3%	0.2459	0.600 [0.270–1.300]
rs1996147	8	22,544,158	G	A	29.3%	17.1%	22.6%	15.3%	41.2%	0.0734	0.500 [0.240–1.030]
rs35900184	8	143,693,411	T	C	31.9%	41.2%	25.1%	34.0%	26.5%	0.2101	1.500 [0.800–2.790]

*42 cases*, *50 controls; 49 males*, *43 females*.

A replication cohort was generated by inclusion of additional cases to the samples from the discovery cohort. No additional controls were included due to lack of availability. The results of association analyses for the two *EGR3* and one *ARC* SNP genotyped for the replication cohort are shown in Tables 4 and 5. In the EU replication study *EGR3* SNP rs1877670 showed a significant association with schizophrenia (p = 0.0078). The *ARC* SNP rs35900184 maintained a significant association with the schizophrenia diagnosis (p = 0.0275) in the replication study. In the AA cohort only the *ARC* SNP rs35900184 demonstrated significance (p = 0.045).

**Table 4:** Replication Phase: Genetic Association Between Schizophrenia and SNPs in *EGR3* and *ARC* in EU Population. Replication EU.

SNP	Chr	Hg18 Position	A1	A2	MAF Controls	MAF Cases	MAF Pooled Controls	MAF Pooled Cases	GMAF	P-value	OR [95% CI]
rs1877670	8	22,546,561	C	T	45.8%	55.6%	42.3%	47.6%	44.3%	0.0078	1.480 [1.110–1.960]
rs1996147	8	22,544,158	G	A	35.5%	32.5%	33.5%	25.0%	41.2%	0.3745	0.880 [0.660–1.170]
rs35900184	8	143,693,411	T	C	22.9%	30.0%	18.0%	28.1%	26.5%	0.0275	1.450 [1.050–2.00]

*386 cases*, *150 controls; 363 males*, *173 females*.

**Table 5:** Replication Phase: Genetic Association Between Schizophrenia and SNPs in *EGR3* and *ARC* in AA Population. Replication AA.

SNP	Chr	Hg18 Position	A1	A2	MAF Controls	MAF Cases	MAF Pooled Controls	MAF Pooled Cases	GMAF	P-value	OR [95% CI]
rs1877670	8	22,546,561	C	T	22.3%	20.5%	29.0%	19.0%	44.3%	0.6716	0.900 [0.520–1.550]
rs1996147	8	22,544,158	G	A	29.3%	25.1%	22.6%	15.3%	41.2%	0.4251	0.810 [0.490–1.340]
rs35900184	8	143,693,411	T	C	31.9%	43.7%	25.1%	34.0%	26.5%	0.0448	1.650 [1.020–2.680]

*185 cases*, *50 controls; 127 males*, *108 females*.

An additional investigation of “Population Controls” from the publicly available 1,000 genomes data was performed [31]. This demonstrated the allele frequency of the four SNPs of interest in presumed controls denoted as “GMAF” in Tables 2–8. These data show that the population controls are in approximately the same allele frequency range as the clinical controls, suggesting that these variants are indeed significantly enriched in the white schizophrenia cases.

**Table 6:** Replication of *ARC* SNP Association with Schizophrenia in Han Chinese Population. Replication CH *ARC*.

SNP	Chr	Hg18 Position	A1	A2	MAF Controls	MAF Cases	GMAF	P-value	OR [95% CI]
rs35900184	8	143,693,411	T	C	17.3%	19.2%	26.5%	0.2933	1.140 [0.910–1.430]

*491 cases*, *491 controls; 525 males*, *457 females*.

**Table 7:** *EGR3* SNP Association Analyses in All Populations Combined. All Samples Combined *EGR3*.

SNP	Chr	Hg18 Position	A1	A2	MAF Controls	MAF Cases	GMAF	P-value	OR [95% CI]
rs1877670	8	22,546,561	C	T	39.7%	44.1%	44.3%	0.1405	1.200 [0.940–1.530]
rs1996147	8	22,544,158	G	A	34.0%	30.1%	41.2%	0.1748	0.840 [0.0650–1.080]

*571 cases*, *200 controls; 490 males*, *281 females*.

**Table 8:** *ARC* SNP Association Analysis in All Populations Combined. All Samples Combined *ARC*.

SNP	Chr	Hg18 Position	A1	A2	MAF Controls	MAF Cases	GMAF	P-value	OR [95% CI]
rs35900184	8	143,693,411	T	C	19.5%	27.2%	26.5%	2.353e-07	1.540 [1.310–1.820]

*1062 cases*, *691 controls; 1015 males*, *735 females*.

### *ARC* Association Study Han Chinese Sample

To test our hypothesis that *ARC*, a direct gene target of *EGR3*, is also a schizophrenia risk gene, we genotyped rs35900184 in a CH population of 491 cases and 491 controls in which *EGR3* had previously demonstrated association with schizophrenia [13]. Table 6 shows that the minor allele of rs35900184 showed no significant difference in frequency between cases and controls (*p* = 0.293) in this CH cohort.

### Combined analysis of *ARC* and *EGR3*

To increase power and to investigate the role of our candidate variants independent of ethnicity we performed a combined analysis of the SNPs (Tables 7 and 8). The *EGR3* SNPs were not significant in the combined group of EU and AA samples (the CH samples were not included as *EGR3* has previously been investigated for associations in this group [13]). However, the *ARC* SNP, rs35900184, was significantly associated with schizophrenia in the combined group of EU, AA, and CH samples (p = 2.5X10^-7^), with a suggested odds ratio of 1.54 (95% confidence interval = 1.31–1.82) (Table 8). We also assessed the heterogeneity of our three candidate SNPs across the study populations using a Breslow-Day test. The results of this indicated significant heterogeneity at SNPs rs1877670 (p = 0.024) and rs35900184 (p = 4.5X10^-3^)(see S6 Table). To address any potential confounds from this heterogeneity we performed a meta-analysis using a random effects model in PLINK (see S7 Table). This resulted in a significant association noted in the ARC SNP only (rs35900184, p = 1.9X10^-4^).

### Variations in the *ARC* Region

A 30 Kb region of chromosome 8 spanning the *ARC* locus was interrogated using HapMap and NCBI dbSNP resources to identify known SNPs and analyze LD in this region. (A similar analysis of the *EGR3* region has previously been published [43].) The *ARC* SNP rs35900184 is not included in either of these public resources. The HapMap project includes 18 SNPs in this region, which have been genotyped in 11 populations (S1A Fig). Genotype data from the CEPH population (Utah residents with ancestry from northern and western Europe, abbreviated CEU) was used to create a linkage disequilibrium map in Haploview, which revealed one haplotype block in this region (S1B Fig). The *ARC* SNP rs35900184 is located outside of the haplotype block in a region that is poorly genotyped in HapMap. Analysis of copy number variation (CNV) domains revealed that *ARC* resides within a CNV region (S1C Fig).

## Discussion

While numerous studies have identified candidate genes and genomic variations linked to risk for psychiatric illnesses, few of them provide an explanation for the role of environment. We have previously proposed that proteins acting in a biological cascade required for memory formation and hippocampal LTD, comprise a pathway that, when disrupted, increases risk for schizophrenia [4, 15, 44]. This pathway, triggered by activity-dependent opening of NMDARs, allows neuronal calcium influx, which acts on CN, a Ca++ activated phosphatase, to activate expression of the IEG transcription factor *EGR3* [19, 21]. The gene *ARC* is a direct target of EGR3 protein [24], and is also required for memory formation and LTD [4]. We therefore hypothesized that, like the preceding proteins in this putative pathway, which have each been implicated in schizophrenia risk, *ARC* should also be a schizophrenia susceptibility gene. Such a collection of risk genes into one functional pathway can address both the polygenic nature of, and environmental contribution to, schizophrenia risk.

In the current study we asked the whether genomic variations in the environmentally-activated IEGs *EGR3* and *ARC* are associated with schizophrenia in two racial populations, EU and AA. We used NGS to comprehensively evaluate the frequency of variations present at the *EGR3* and *ARC* loci in both cases and controls. This allowed identification of multiple types of polymorphisms, including rare variants, providing a much greater depth of investigation than a classical SNP genotyping approach, which is limited to detection of SNPs occurring at >10% frequency in the population. We hypothesized that this would be a more effective way to select variations for genotyping than choosing known SNPs randomly across the genomic region for an association study. It also allowed rapid and broad comparison of variation frequencies among racial populations. We indicate the concordance in MAF of our candidate variants in Tables 2 and 3. This demonstrates that the pooled sequencing approach is highly comparable to the results obtained on the same individuals via genotyping.

Our major findings were that *EGR3* SNP rs1877670 was associated with schizophrenia in our population of EU cases and controls (p = 0.0078; Table 4). And the *ARC* SNP rs35900184 displayed association with schizophrenia risk in both the EU and the AA populations (p = 0.0275 and p = 0.0448, respectively; Tables 4 and 5). Further, although *ARC* SNP rs35900184 did not show statistically significant association with schizophrenia in the CH population alone (Table 6), when all three racial groups were combined to increase statistical power, rs35900184 revealed a strongly significant association with schizophrenia (p = 2.353 x 10^−7^; OR [95% CI] = 1.54 [1.310–1.820]; Table 8). These findings support our hypothesis of a biological pathway involving memory, hippocampal LTD, and schizophrenia susceptibility [4, 15, 44], by demonstrating that *ARC*, the downstream target of proteins in the pathway that are associated with risk for schizophrenia, is itself associated with risk for this illness.

To our knowledge, this is the first study examining association between a SNP in *ARC* and schizophrenia. It is also the first study reporting associations between SNPs in *EGR3* and schizophrenia in an AA population. Recent high-profile studies of rare copy number variations (CNVs) and *de novo* variations enriched in schizophrenia patients have identified the importance of proteins that complex with ARC protein in the post-synaptic density in the risk for schizophrenia [28–30, 45]. Small *de novo* mutations in genes encoding proteins that associate with ARC are overrepresented in schizophrenia patients versus controls [30]. And exome sequencing has identified rare disruptive mutations involving proteins that associate with ARC are more prevalent in schizophrenia patients than controls [29]. However, these large-scale genome-wide studies did not identify associated variations in the *ARC* gene itself. Notably, some of these were exome studies, so would not have included the regulatory region in which the ARC SNP rs35900184 resides.

To date, three case-control and one family-based study have identified significant associations between SNPs in *EGR3* and schizophrenia [11–13, 41]. An additional family study revealed nominally significant transmission disequilibrium for *EGR3* [11]. All of these studies were in Asian populations, including Korean, Japanese, and CH. In all three case-control studies the same SNP, rs35201266, demonstrated association with schizophrenia [11–13]. In the CH family study rs1996147 and rs3750192 showed association with schizophrenia, as did the haplotype rs3750192-rs35201266 [41]. Other case-control studies in the Japanese and CH populations failed to identify significant associations between SNPs in *EGR3* and schizophrenia [46–48], though only one of these three studies included rs35201266 [46].

*EGR3* SNP rs35201266 has also been associated with prefrontal hemodynamic response to a verbal fluency test in both control and schizophrenia subjects of Japanese ethnicity, suggesting a relationship between *EGR3 and* cognitive function in a brain region implicated in schizophrenia [49]. rs35201266 resides in the single intron of *EGR3*, suggesting a possible functional role in *EGR3* mRNA expression or post-transcriptional processing. *In vitro* analysis of this SNP demonstrated a potential functional effect on transcription [11]. However, we did not genotype rs35201266 in our samples as our NGS results demonstrated a Δ MAF of 4.3% in the EU group and 3.2% in the AA group for this SNP, which did not meet our cut-off of scoring > 5% in at least one group.

Only one study examining *EGR3* associations in schizophrenia in a EU population has been reported. Although that study, which focused on circadian rhythm-related genes, failed to identify an association between SNPs in *EGR3* and schizophrenia, it did report a suggestive association between *EGR3* and bipolar I disorder (p = 0.017), though this did not survive control for multiple comparisons [42]. Nominal associations were also found between two linked *EGR3* SNPs, rs10104039 and rs10095121, and child bipolar I disorder in a family based association study of primarily EU participants (88% EU; p = 0.027 and p = 0.028) [43]. Numerous findings, including reports from genome wide association studies (GWAS), indicating shared genetic influences on both bipolar disorder and schizophrenia, highlight the potential relevance of these findings for our study [50–52].

In addition to the genetics findings, post mortem studies have reported reduced *EGR3* mRNA levels in schizophrenia patients’ brains [11, 14], and a biomedical informatics study identified *EGR3* as the central gene in a network of microRNAs and transcription factors implicated in schizophrenia pathogenesis [53]. *EGR3* also resides in a copy number variation (CNV)-containing region of the chromosome [43], and CNVs have demonstrated a high correlation with schizophrenia and other psychiatric illnesses [54].

The 8p chromosomal region, and indeed the specific location of *EGR3* at 8p21.3, have been repeatedly associated with schizophrenia susceptibility, specifically in the AA population [55–58]. The importance of this region in schizophrenia is also supported by meta- analysis of 32 Genome Wide Linkage studies [59] and rank-based genome scan meta-analysis from 20 schizophrenia genome scans [60]. Although our study did not demonstrate association between schizophrenia and *EGR3* SNPs rs1877670 or rs1996147 in the AA cohort, the limited number of available AA DNA samples led to the study being insufficiently powered to detect variations of low effect size. Interestingly, rates of schizophrenia have been reported to be up to three times higher in AA than EU individuals, which may be due to differences in both genetic and environmental causes [61].

The major limitation of our study was the number of samples available, particularly in the AA group. Because of this, the DNA samples used for the discovery NGS study were included in the validation phase. This was done for two reasons. First, the initial discovery phase used a pooling approach that allowed us to cost-effectively sequence the region with a very high copy number (providing a high level of accuracy), but did not discern individual genotype results (only an average for the pool). Follow-up genotyping was therefore necessary to conduct association analyses. Second, the number of additional available samples was insufficient to power an independent study. Inclusion of the original discovery set of DNAs in the genotype analysis allowed us to increase our number of samples, which was still of a size that limited the power of the study.

Lastly, it is important to note that the identified variants do not immediately suggest any potential biological function; therefore, it is possible that they are closely linked with neighboring variants that may influence function of the underlying proteins. For example, rs1877670 falls within the 3’ UTR of EGR3, but doesn’t overlap with any known regulatory regions in the gene, rs1996147 is located ~143kb downstream of EGR3 in the current build of the human genome (GRCh37), and rs35900184 falls within an intron between exons 2 and 3 of ARC and doesn’t overlap any known regulatory regions. Therefore the putative functional role of these variants is in need of additional investigation.

## Conclusions

We report that the *EGR3* SNP rs1877670 is associated with schizophrenia in an EU population. In addition, the *ARC* SNP rs1877670 shows association in both the EU and AA populations, and is highly significant in a combined group of EU, AA, and CH populations. These findings support our hypothesis that dysfunction of genes in the biological pathway of NMDA-receptors to *EGR3* regulation of *ARC* may increase schizophrenia susceptibility. This pathway represents a molecular link between environment and the post-synaptic density proteins recently implicated in schizophrenia risk by CNV and GWAS studies [28–30], and may provide insight into the cognitive deficits characteristic of this illness.

## Supporting Information

S1 Fig NCBI and HapMap Data on Variations in the *ARC* Region. A 30 Kb region of chromosome 8, spanning from 143676124 to 143706123, which contains the *ARC* gene, was interrogated. **a.** HapMap data version 28 includes 18 SNPs genotyped in this region. Graphics below the chromosome line indicate percentages of each allele for the SNP (indicated in blue versus red) for each racial-ethnic population genotyped; rs numbers below the HapMap results show the 126 SNPs reported in this region on NCBI B36, dbSNP. They do not include rs35900184. Yellow highlight indicates the location of the *ARC* gene. **b.** Linkage disequilibrium map created in Haploview using the CEU genotype data for SNPs in this region identified a single haplotype block. *ARC* (chr8: 143,689,412 to 143,692,835) is located between SNP 16 (rs13260813 A/C chr8 143,689,397) and SNP 18 (rs28473387 C/T chr8 143,704,475), in a region that is poorly genotyped in HapMap CEU data. *ARC* SNP: rs35900184 (8:143,690,413), is located between SNP 16 (rs13260813 A/C; chr8 143,689,397) and SNP 17 (rs10097505 A/G; chr8 143,691,186, an *ARC* SNP) in the middle of a region of poor LD. **c.** The *ARC* gene resides in a copy number variation region.
(PDF)

S1 Table Primer Sequences Used to Generate PCR Amplicons from Pooled DNAs for Next Generation Sequencing. (XLSX)

S2 Table Variations Identified in *EGR3* by Next Generation Sequencing from EU DNA Pools. Bold type indicates SNPs selected for follow-up genetic association analysis.
(XLSX)

S3 Table Variations Identified in *EGR3* by Next Generation Sequencing from AA DNA Pools. Bold type indicates SNPs selected for follow-up genetic association analysis.
(XLSX)

S4 Table Variations Identified in *ARC* by Next Generation Sequencing from EU DNA Pools. Bold type indicates SNPs selected for follow-up genetic association analysis.
(XLSX)

S5 Table Variations Identified in *ARC* by Next Generation Sequencing from AA DNA Pools. Bold type indicates SNPs selected for follow-up genetic association analysis.
(XLSX)

S6 Table Breslow-Day test for heterogeneity between AA, EU, and CH populations. These results suggest that rs1877670 and rs35900184 SNPs are significantly heterogeneous according to this test. Significant p-values are indicated in bold red text.
(XLSX)

S7 Table Meta-analysis of combined populations. Meta-analyses were conducted under both a fixed- and random-effect model. Based on the Breslow-Day results in S6 Table, the fixed-effect model is not the most appropriate test for these data. The random-effect model results are presented in the column headed “P(R)”. Significant p-value is indicated in bold red text.
(XLSX)
