# Supplementary material for: Association of SNPs in EGR3 and ARC with Schizophrenia Supports a Biological Pathway for Schizophrenia Risk
Source: PLoS One. 2015 Oct 16;10(10):e0135076. doi: 10.1371/journal.pone.0135076 (PMC4608790; doi:10.1371/journal.pone.0135076)
Supplement: S1 Fig — A 30 Kb region of chromosome 8, spanning from 143676124 to 143706123, which contains the ARC gene, was interrogated. a. HapMap data version 28 includes 18 SNPs genotyped in this region. Graphics below the chromosome line indicate percentages of each allele for the SNP (indicated in blue versus red) for each racial-ethnic population genotyped; rs numbers below the HapMap results show the 126 SNPs reported in this region on NCBI B36, dbSNP. They do not include rs35900184. Yellow highlight indicates the location of the ARC gene. b. Linkage disequilibrium map created in Haploview using the CEU genotype data for SNPs in this region identified a single haplotype block. ARC (chr8: 143,689,412 to 143,692,835) is located between SNP 16 (rs13260813 A/C chr8 143,689,397) and SNP 18 (rs28473387 C/T chr8 143,704,475), in a region that is poorly genotyped in HapMap CEU data. ARC SNP: rs35900184 (8:143,690,413), is located between SNP 16 (rs13260813 A/C; chr8 143,689,397) and SNP 17 (rs10097505 A/G; chr8 143,691,186, an ARC SNP) in the middle of a region of poor LD. c. The ARC gene resides in a copy number variation region. (PDF) [file pone.0135076.s001.pdf]

S1 Fig.

(A)

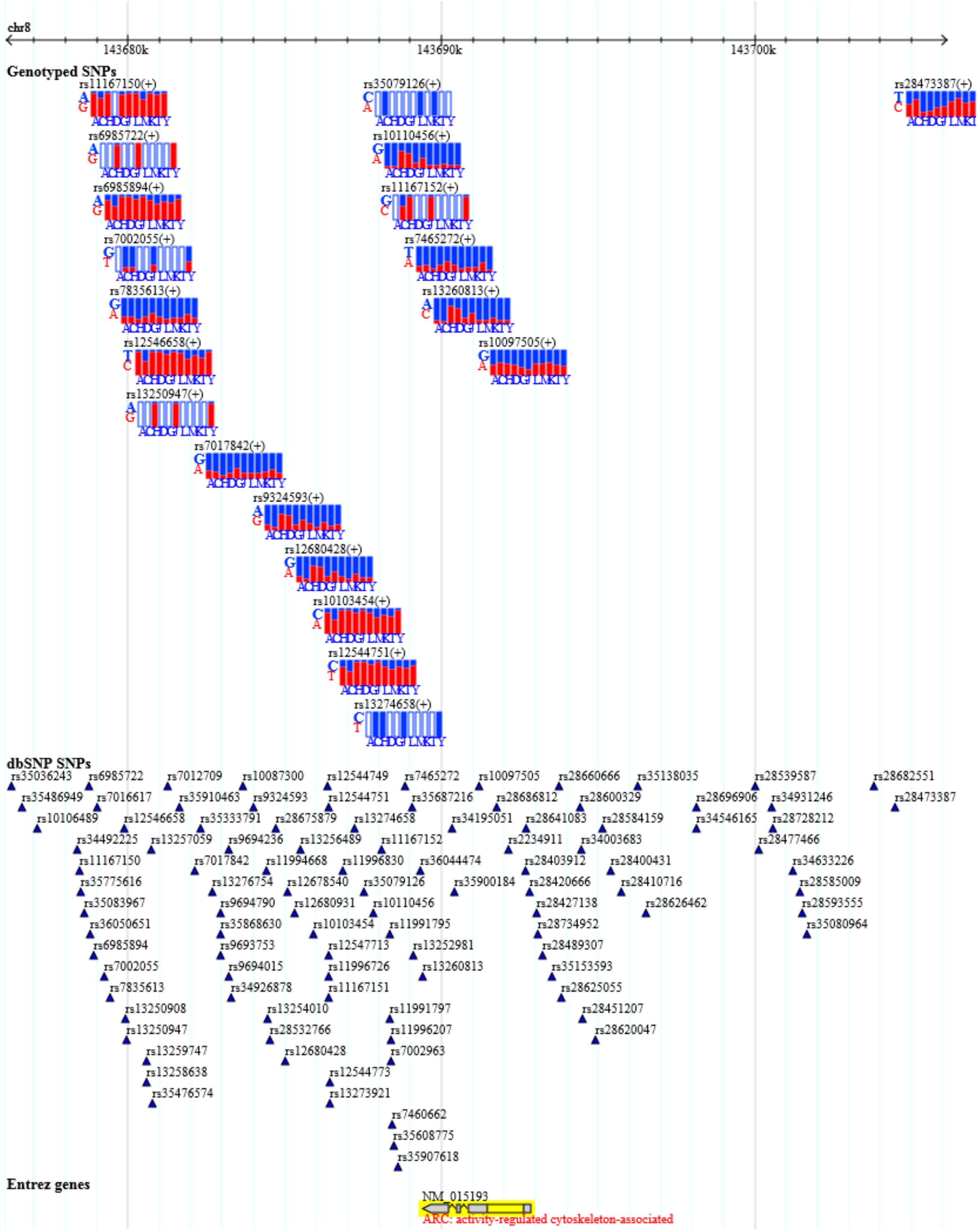

chr8
143680k
143690k
143700k
Genotyped SNPs
rs11167150(+)
rs6985722(+)
rs6985894(+)
rs7002055(+)
rs7835613(+)
rs12546658(+)
rs13250947(+)
rs7017842(+)
rs9324593(+)
rs12680428(+)
rs10103454(+)
rs12544751(+)
rs13274658(+)
rs35079126(+)
rs10110456(+)
rs11167152(+)
rs7465272(+)
rs13260813(+)
rs10097505(+)
rs28473387(+)
dbSNP SNPs
Entrez genes
NM_015193
ARC: activity-regulated cytoskeleton-associated

(B)

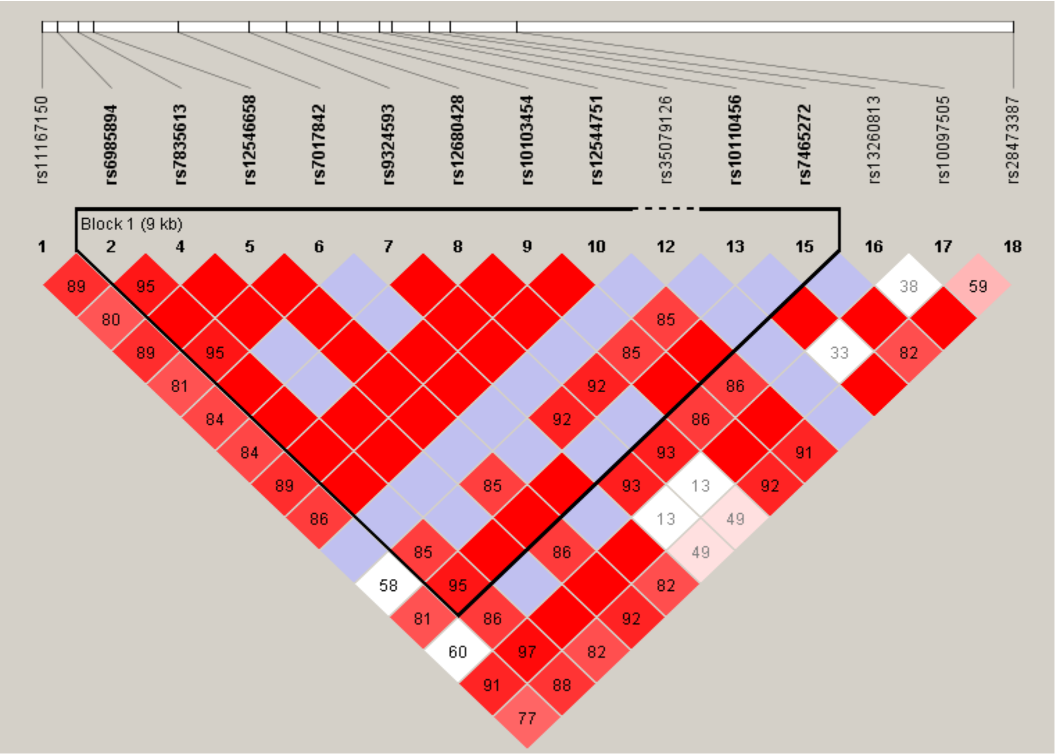

(C)

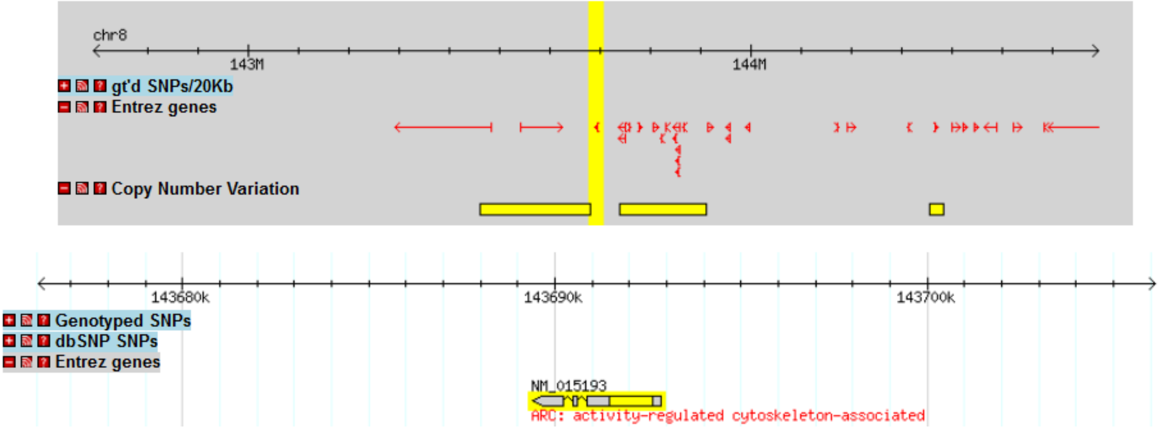

NCBI and HapMap data on variations in the *ARC* region.
Results of interrogation of a 30 Kb region of chromosome 8, spanning from 143676124 to 143706123, which contains the *ARC* gene was interrogated. **a.** HapMap data version 28 includes 18 SNPs genotyped in this region. Graphics below the chromosome line indicate percentages of each allele for the SNP (indicated in blue versus red) for each racial-ethnic population genotyped; rs numbers below the HapMap results show the 126 SNPs reported in this region on NCBI B36, dbSNP. They do not include rs35900184. Yellow highlight indicates the location of the *ARC* gene. **b.** Linkage disequilibrium map created in Haploview using the CEU genotype data for SNPs in this region identified a single haplotype block. *ARC* (chr8: 143,689,412 to 143,692,835) is located between SNP 16 (rs13260813 A/C chr8 143,689,397) and SNP 18 (rs28473387 C/T chr8 143,704,475), in a region that is poorly genotyped in HapMap CEU data. *ARC* SNP: rs35900184 (8:143,690,413), is located between SNP 16 (rs13260813 A/C; chr8 143,689,397) and SNP 17 (rs10097505 A/G; chr8 143,691,186, an *ARC* SNP) in the middle of a region of poor LD. **c.** The *ARC* gene resides in a copy number variation region.
